# Effects of essential oil blend supplementation on growth performance, nutrient digestibility, immune response, villus histology and cecal microbiota in broilers

**DOI:** 10.5194/aab-69-205-2026

**Published:** 2026-03-30

**Authors:** Nisar Muhammad Khan, Muhammad Tahir, Shabana Naz, Rifat Ullah Khan, Rasha Alonaizan, Rasha K. Al-Akeel, Ala Abudabos, Raed M. Al-Atiyat, Ihteshamul Haq, Ibrahim A. Alhidary

**Affiliations:** 1 Department of Animal Nutrition, Faculty of Animal Husbandry and Veterinary Sciences, The University of Agriculture, Peshawar, Pakistan; 2 Department of Zoology, Government College University, Faisalabad, Pakistan; 3 Physiology Lab, College of Veterinary Sciences, Faculty of Animal Husbandry and Veterinary Sciences, The University of Agriculture, Peshawar, Pakistan; 4 Department of Zoology, College of Science, King Saud University, P.O. Box 2455, Riyadh 11451, Saudi Arabia; 5 Department of Food and Animal Sciences, College of Agriculture, Tennessee State University, Nashville, TN 37209, USA; 6 Molecular Genetics, Breeding and Biotechnology, Animal Sci. Dep., Agriculture Faculty, Mutah University, Karak, Jordan; 7 Institute of Biotechnology and Genetic Engineering, The University of Agriculture, Peshawar, Pakistan; 8 Department of Animal Production, College of Food and Agriculture Sciences, King Saud University, Riyadh, Saudi Arabia

## Abstract

This study examined the effects of varying essential oil (EO) blend levels on growth performance, nutrient digestibility, immune response, villus histology and cecal microbiota in Hubbard male broiler chicks. Five groups of male chicks ( n= 1000) received diets with incremental EO blend levels: 0, 0.5, 1, 1.5, and 2 mL kg^−1^ feed. Results showed no significant initial differences in feed intake or body weight gain. However, by week 3, the group supplemented with 2 mL kg^−1^ exhibited significantly higher feed intake and weight gain ( p<0.05 ), sustaining these trends throughout weeks 4 and 5. The group supplemented with 2 mL kg^−1^ achieved a better feed conversion ratio (FCR) in weeks 4 and 5, culminating in the most favorable overall FCR across the trial period ( p= 0.04). Digestibility assessments revealed that supplementation of 2 mL kg^−1^ resulted in the highest dry matter, gross energy, ether extract, and crude protein digestibility levels ( p<0.05 ), suggesting enhanced nutrient absorption. Immunological results indicated significantly elevated antibody titers in the group supplemented with 2 mL kg^−1^ against Newcastle disease (ND), infectious bronchitis (IB), and infectious bursal disease (IBD). The group supplemented with 2 mL kg^−1^ consistently achieved the highest antibody levels, notably with ND titers on day 35 ( p= 0.04). These findings suggest that a 2 mL kg^−1^ EO blend supplementation optimizes growth, nutrient utilization, and immune response, offering a natural alternative to promote broiler health and performance.

## 1 Introduction

Recent studies have highlighted the growing shift toward natural alternatives to antibiotic growth promoters (AGPs) in poultry production, demonstrating the potential of plant-based, microbial, and essential-oil-derived additives to improve performance, immunity, and gut health (Ullah et al., 2022; Alqahtani et al., 2024; Dablool et al., 2024). These findings reinforce the viability of phytogenic solutions as sustainable replacements for conventional AGPs in intensive broiler systems (Rahman et al., 2017; Hassan et al., 2023; Su et al., 2023; Malik et al., 2024; Rasool et al., 2024; Wang et al., 2025).

Essential oils (EOs) have received special attention due to their established antimicrobial, antioxidant, anti-inflammatory, and digestive-stimulatory properties. Several studies demonstrate their benefits for growth performance, nutrient utilization, and gut integrity in broilers (Khan et al., 2023; Chen et al., 2023). While many investigations have focused on single-oil supplementation – mainly thymol, carvacrol, cinnamon, oregano, or garlic – emerging evidence suggests that synergistic blends may produce stronger physiological and metabolic effects by combining multiple active compounds (Abd El-Hack et al., 2022). For example, blends containing thymol, carvacrol, or eugenol have been shown to enhance digestive enzyme secretion, modulate cecal microbiota, and reduce pathogenic bacteria more effectively than individual oils (Mathlouthi et al., 2012; Khattak et al., 2014; Hafeez et al., 2020a).

However, most previous studies on EO blends relied on two-component combinations (e.g., thymol + carvacrol) or used low inclusion levels, making it difficult to identify optimal supplementation doses for maximizing growth, digestibility, and immune competence. Moreover, the biological responses to EO supplementation can vary extensively with dose, botanical source, extraction technology, and bird age or health status (Reisinger et al., 2011; Khattak et al., 2014). High doses may depress feed intake or compromise palatability (Zhang et al., 2019), whereas insufficient doses often fail to elicit measurable improvements.

Thus, this research fills a critical gap by determining the optimal inclusion level of a complex, multi-molecule essential oil blend – a novel approach that extends beyond the single-oil and dual-oil strategies commonly reported in the literature – and by validating its effects across key physiological, immunological, and microbiological parameters relevant to antibiotic-free poultry production.

## 2 Materials and methods

In this research trial, 1000 day-old male broiler (Hubbard) chicks were divided into five dietary treatment groups (O, A, B, C, and D), with each treatment replicated five times. Each replicate consisted of 40 birds housed in a single pen, and therefore the pen (not the individual bird) was considered the experimental unit in the statistical analysis. The study followed a completely randomized design (CRD). The chicks were housed in a comfortable, controlled environment with regulated temperature, ventilation, and humidity on clean wood shavings as bedding material. The bedding was maintained at a depth of approximately 5 cm to ensure proper moisture absorption and comfort. Each pen measured 1.5 m^2^, providing a stocking density of 30 kg m^−2^. A 23L : 1D lighting schedule was implemented to optimize growth performance. The birds were fed a commercial starter diet from day 1 to 21 and a finisher diet from day 22 to 35, formulated to meet the nutrient requirements of broilers according to NRC (1994) guidelines as shown in Table 1. The chemical composition of feed samples was analyzed for crude protein, ether extract, crude fiber, ash, calcium, and phosphorus using the standard methods of the Association of Official Analytical Chemists (AOAC, 2005). Feed and water were provided equally to all groups throughout the trial. All birds were vaccinated against infectious bronchitis virus (IBV), Newcastle disease virus (NDV), and infectious bursal disease virus (IBDV) according to a standard commercial broiler vaccination program. The vaccination schedule was as follows: IBV and NDV (live vaccines) were administered via drinking water on day 5 and day 18 of age, and the IBDV vaccine was administered on day 12 and day 22.

**Table 1:** Ingredients and feed composition of broilers on dry matter basis.

Percent constituents	Starter to grower	Finisher phase
(%)	phase (1–21 d)	(22–35 d)
Maize (yellow)	59.0	58.0
Soybean meal (48 %)	37.0	37.5
Limestone	1.4	1.6
Dicalcium phosphate	1.6	1.65
Salt (NaCl)	0.4	0.5
DL-Methionine	0.15	0.25
L-Lysine	0.85	0.90
L-Threonine	0.55	0.60
L-Tryptophan	0.15	0.20
Vitamin and mineral premixes^*^	0.5	0.50
Chemical analysis		
Crude protein (%)	22.5	21.0
Metabolizable energy (kcal kg^−1^)	2850	2950
Crude fiber (%)	3.0	3.5
Crude fat (%)	3.1	3.4
Lysine (%)	1.3	1.3
Calcium (%)	0.95	0.85
Methionine + cysteine (%)	0.82	0.72
Threonine (%)	0.9	0.6
Available phosphorus (%)	0.45	0.45
Sodium (%)	0.2	0.16
Chloride (%)	0.2	0.16

^*^ Provided per kg of diet: vitamin A, 15000 IU; vitamin E, 15 IU; vitamin D3, 2500 IU; thiamine, 2 mg; vitamin K3, 2.5 mg; pyridoxine, 5 mg; riboflavin, 5.5 mg; folic acid, 0.7 mg; vitamin B12, 0.12 mg; pantothenic acid, 15 mg; nicotinic acid, 45 mg; biotin, 0.25 mg; iron, 95 mg; copper, 10 mg; zinc, 100 mg; manganese, 100 mg; iodine, 0.5 mg; selenium, 0.3 mg.

Blood samples for antibody titer determination were collected on days 21 and 35 of age. Therefore, the first sampling point (day 21) corresponded to the post-primary immune response phase, whereas the second sampling point (day 35) reflected the booster response following the second vaccination. This timeline allows proper interpretation of the humoral immune response dynamics relative to vaccination.

### 2.1 Bird management and trial arrangement

The chicks were raised under uniform management conditions, including a temperature-controlled environment that followed a standard brooding schedule: 33 °C during the first week, gradually reduced to 23 °C by the end of the third week. Humidity levels were maintained between 50 % and 60 %. Adequate ventilation was provided using an automated ventilation system. Each replicate (pen) housed 30 birds, allowing sufficient space for movement and minimizing stress. Feed and water were made available ad libitum, and feeders and drinkers were cleaned daily to maintain hygiene.

A blend of essential oils (Crina^®^ Poultry Plus, DSM Nutritional Products Ltd., Kaiseraugst, Switzerland; product code 10286180, liquid formulation) was supplemented in feed at different levels for each experimental group, as follows:

Composition of essential oil blend (per 1000 mL):

- Oregano oil: 35 000 mg, – Cinnamon oil: 100 000 mg, – Thyme oil: 30 000 mg,
- Capsicum oleoresin: 20 000 mg, – Garlic powder: 10 000 mg.

To ensure uniform distribution, a two-step mixing procedure was applied. First, the required quantity of the liquid essential oil blend was diluted in a small portion of feed (approximately 5 kg) to form a premix. This premix was manually mixed for 5 min to allow complete absorption of the liquid into the carrier feed particles. Subsequently, the premix was incorporated into the remaining feed in a horizontal mechanical mixer and blended for 15 min to achieve homogeneity.

### 2.2 Distribution of birds and grouping

The broiler chicks were grouped as follows based on essential oil supplementation mixed into their feed:

- Group O: Control group, provided with only a basal diet and no essential oil supplementation,
- Group A: Basal diet mixed with 0.5 mL of essential oil blend per kg of feed,
- Group B: Basal diet mixed with 1 mL of essential oil blend per kg of feed,
- Group C: Basal diet mixed with 1.5 mL of essential oil blend per kg of feed,
- Group D: Basal diet mixed with 2 mL of essential oil blend per kg of feed.

This grouping was intended to analyze the effects of essential oil levels in feed on the growth performance and health of broilers.

### 2.3 Growth performance

Weekly feed intake was calculated by measuring the difference between the amount of feed offered and the feed remaining (rejected) at the end of each week. The weekly average body weight gain was calculated by recording the difference between the total weight at the end of each week and the initial weight at the beginning of that week. Feed conversion ratio (FCR) was calculated weekly to determine the efficiency of feed utilization. This was done by dividing the total feed consumed by the total weight gained for that week.

Measurements of growth performance variables (feed intake, body weight, and FCR) and antibody titers were recorded by personnel who were not informed of the treatment allocations of each group. Pen labels did not indicate treatment codes, ensuring that assessors remained unaware of dietary treatments during data collection. This blinding procedure minimized observer bias in both growth and immunological measurements.

### 2.4 Determination of apparent nutrient digestibility 

On day 31, five birds per replicate, selected based on similar body weight to ensure uniformity, were moved to metabolic cages for a 4 d fecal collection period. Feed intake and fecal output were recorded daily to evaluate nutrient digestibility. To determine digestibility using the marker method, titanium dioxide (0.5 %) was included in the experimental diets as an indigestible marker. Carcasses were dissected, and the ileum was collected and preserved for chemical analysis. After freeze drying, dry matter, crude protein, ether extract, crude fiber, and apparent metabolizable energy levels in the feed and ileal digesta samples were measured following the methodology of Hafeez et al. (2020b). Gross energy (GE) was measured using an adiabatic bomb calorimeter; ether extract (EE) was determined by Soxhlet extraction; and crude protein (CP) was analyzed using the Kjeldahl method. Titanium dioxide concentration in feed and digesta was analyzed using UV spectrophotometry, and apparent ileal digestibility coefficients were calculated based on the ratio of titanium dioxide in feed and ileal digesta.

1 $$\begin{array}{rl} & \mathrm{Apparent}\;\mathrm{ileal}\;\mathrm{digestibility}(\%)=100\\ & -\frac{\mathrm{conc}.\;\mathrm{of}\;\mathrm{marker}\;\mathrm{in}\;\mathrm{feed}}{\mathrm{conc}.\;\mathrm{of}\;\mathrm{marker}\;\mathrm{in}\;\mathrm{digesta}\;}\\ & \times \frac{\mathrm{conc}.\;\mathrm{of}\;\mathrm{nutrient}\;\mathrm{in}\;\mathrm{digesta}}{\mathrm{conc}.\;\mathrm{of}\;\mathrm{nutrient}\;\mathrm{in}\;\mathrm{feed}}\times 100 \end{array}$$

### 2.5 Humoral immune response

To assess immunity against ND, IB, and IBD, representative blood samples (2–3 mL each) were collected from five to six birds per pen using sterile syringes on days 7, 14, 21, 28, and 35. The samples were transferred to clot tubes and allowed to sit at 27–38 °C for 1–2 h to obtain serum. Once separated, the serum was transferred to labeled collection tubes, tightly sealed, and stored at 4 °C for transport to the laboratory. There, serum samples were centrifuged, diluted, and analyzed using HA : HI and ELISA tests to determine antibody titers against ND, IB, and IBD.

### 2.6 Villus histomorphology

The villus histomorphology of broilers was assessed on day 35 of the experiment by randomly selecting two birds per replicate. The birds were euthanized, and a segment of the ileum was carefully excised approximately 2 cm proximal to the ileocecal junction. The ileal tissues were fixed in a 10 % neutral buffered formalin solution, processed, and embedded in paraffin. Thin sections (4–5 µ m) were stained with hematoxylin and eosin (H&E) for microscopic examination to measure villus height, crypt depth, and the villus-to-crypt ratio. For each bird, multiple well-oriented villi and associated crypts were measured, and the mean value per bird was calculated. The two bird means in each replicate (pen) were then averaged to obtain a single value per replicate. These replicate means were used as the experimental unit for statistical analysis, consistent with the pen-based experimental design.

### 2.7 Cecal microbiota enumeration

On day 35 of the trial, cecal contents were collected from two randomly selected broilers per replicate to assess the bacterial population, specifically *Salmonella* spp., *Escherichia coli*, and *Lactobacillus* spp. Approximately 1 g of the cecal sample was homogenized in buffered peptone water ( 1:10 dilution) and subsequently plated onto selective agar media for bacterial enumeration. *Salmonella* spp. were isolated using xylose lysine deoxycholate (XLD) agar, while *Escherichia* *coli* was cultured on MacConkey agar. *Lactobacillus* spp. were grown on de Man, Rogosa, and Sharpe (MRS) agar at 37 °C under 5 % CO_2_ for 24 h. Bacterial colonies were counted using a colony counter and expressed as log_10_ colony-forming units per gram (CFU g^−1^).

### 2.8 Statistical analysis

All data were analyzed using a one-way analysis of variance (ANOVA) under a completely randomized design using SPSS software (version 21.0; IBM Corp., Armonk, NY, USA). Treatment was considered the fixed effect; and pen served as the experimental unit for all performance, digestibility, immune response, and intestinal morphology parameters. When the overall F test indicated significance, differences among treatment means were separated using Tukey's honestly significant difference (HSD) test. Statistical significance was declared at P<0.05 . Additionally, orthogonal polynomial contrasts (linear and quadratic) were performed to evaluate the effect of increasing levels of essential oil supplementation on the measured parameters.

## 3 Results

Feed intake did not differ among treatments during week 2 ( P= 0.75). Significant differences were observed from week 3 onward (week 3: P= 0.036; week 4: P= 0.044; week 5: P= 0.020; overall: P= 0.040; Table 2). During weeks 3–5 and overall, Group D differed from Group O and Group A, while Groups B and C showed intermediate responses with partial statistical overlap. Linear and quadratic contrasts were significant during these periods ( P≤0.03 ), indicating a graded treatment effect.

**Table 2:** Effect of different levels of blend of essential oil on feed intake (g) in broiler chicks.

	Week 2		Week 3		Week 4		Week 5		Overall	
Group 0	437.33 ± 3.21		729.67^c^ ± 2.08		975.80^c^ ± 1.16		991.10^c^ ± 2.15		3133^c^ ± 4.53	
Group A	436.67 ± 2.85		737.01^c^ ± 4.98		985.50^c^ ± 3.46		1012.77^c^ ± 4.88		3172^c^ ± 7.50	
Group B	417.00 ± 4.17		748.33^bc^ ± 7.57		1014.8^bc^ ± 4.50		1047.6^bc^ ± 4.58		3228^bc^ ± 6.804	
Group C	434.33 ± 5.17		780.67^b^ ± 2.14		1021.8^b^ ± 3.96		1080.3^b^ ± 3.23		3317^b^ ± 5.53	
Group D	424.32 ± 3.17		821.66^a^ ± 3.14		1085.4^a^ ± 2.96		1122.8^a^ ± 2.23		3454^a^ ± 5.53	
P value	0.75		0.036		0.044		0.02		0.04	
Orthogonal contrast	Linear	0.33	Linear	0.03	Linear	0.01	Linear	0.02	Linear	0.01
	Quadratic	0.11	Quadratic	0.01	Quadratic	0.01	Quadratic	0.01	Quadratic	0.02

Means having different superscript in columns are significantly different ( p<0.05 ). Group O: Control group, provided with only a basal diet and no essential oil supplementation; Group A: basal diet mixed with 0.5 mL of essential oil blend per kg of feed; Group B: basal diet mixed with 1 mL of essential oil blend per kg of feed; Group C: basal diet mixed with 1.5 mL of essential oil blend per kg of feed; Group D: basal diet mixed with 2 mL of essential oil blend per kg of feed.

**Table 3:** Effect of different levels of blend of essential oil on body weight gain (g) in broiler chicks.

	Week 2		Week 3		Week 4		Week 5		Overall	
Group 0	185 ± 2.88		272^b^ ± 4.80		462^c^ ± 4.66		871^c^ ± 3.58		1786^bc^ ± 11.24	
Group A	183 ± 1.45		247^c^ ± 3.83		493^b^ ± 4.01		930^b^ ± 2.85		1852^b^ ± 9.68	
Group B	179 ± 4.64		263^bc^ ± 2.08		464^c^ ± 4.69		961^ab^ ± 3.23		1862^ab^ ± 11.86	
Group C	193 ± 3.44		283^a^ ± 2.62		533^a^ ± 3.53		976^a^ ± 2.58		1981^ab^ ± 9.38	
Group D	195 ± 2.34		290^a^ ± 1.64		540^a^ ± 4.58		983^a^ ± 3.45		2008^a^ ± 9.38	
P value	0.09		0.01		0.03		0.02		0.01	
Orthogonal contrast	Linear	0.22	Linear	0.02	Linear	0.01	Linear	0.01	Linear	0.01
	Quadratic	0.54	Quadratic	0.03	Quadratic	0.01	Quadratic	0.01	Quadratic	0.04

Means having different superscript in columns are significantly different ( p<0.05 ). Group O: Control group, provided with only a basal diet and no essential oil supplementation; Group A: basal diet mixed with 0.5 mL of essential oil blend per kg of feed; Group B: basal diet mixed with 1 mL of essential oil blend per kg of feed; Group C: basal diet mixed with 1.5 mL of essential oil blend per kg of feed; Group D: basal diet mixed with 2 mL of essential oil blend per kg of feed.

**Table 4:** Effect of different levels of blend of essential oil on feed conversion ratio (FCR) in broiler chicks.

	Week 2		Week 3		Week 4		Week 5		Overall	
Group 0	2.35 ± 0.04		2.68 ± 0.14		2.13^b^ ± 0.06		1.12^a^ ± 0.03		1.76^a^ ± 0.01	
Group A	2.37 ± 0.14		2.75 ± 0.10		1.91^bc^ ± 0.17		1.07^b^ ± 0.08		1.74^a^ ± 0.04	
Group B	2.35 ± 0.02		2.63 ± 0.21		2.31^a^ ± 0.04		1.08^b^ ± 0.03		1.71^a^ ± 0.01	
Group C	2.27 ± 0.04		2.51 ± 0.06		1.97^bc^ ± 0.07		1.07^b^ ± 0.05		1.69^b^ ± 0.01	
Group D	2.28 ± 0.03		2.55 ± 0.02		1.83^c^ ± 0.07		1.06^bc^ ± 0.05		1.63^bc^ ± 0.01	
P value	0.78		0.38		0.04		0.03		0.04	
Orthogonal contrast	Linear	0.57	Linear	0.13	Linear	0.01	Linear	0.01	Linear	0.01
	Quadratic	0.88	Quadratic	0.21	Quadratic	0.01	Quadratic	0.01	Quadratic	0.04

Means having different superscript in columns are significantly different ( p <0.05). Group O: Control group, provided with only a basal diet and no essential oil supplementation; Group A: basal diet mixed with 0.5 mL of essential oil blend per kg of feed; Group B: basal diet mixed with 1 mL of essential oil blend per kg of feed; Group C: basal diet mixed with 1.5 mL of essential oil blend per kg of feed; Group D: basal diet mixed with 2 mL of essential oil blend per kg of feed.

**Figure 1:**
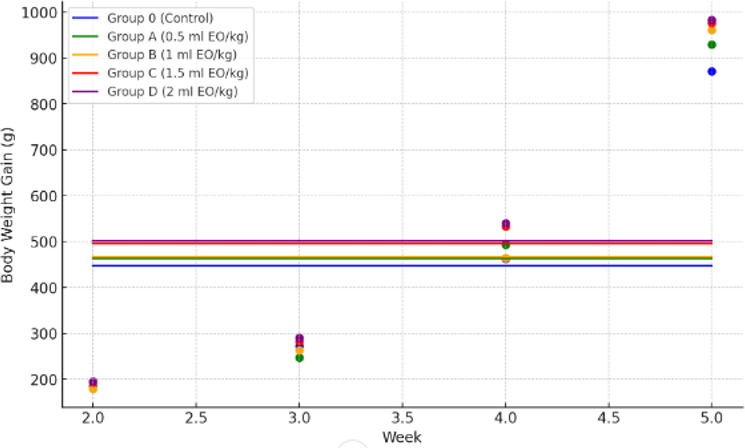
Weekly body weight gain of broilers in different treatment groups (Control, 0.5, 1, 1.5, and 2 mL kg^−1^ essential oil). Data points represent mean values per week, connected by lines for visualization of growth trends.

Body weight gain did not differ during week 2 ( P= 0.09). Differences emerged during weeks 3–5 and overall ( P≤0.03 ), where Group D showed greater gains than Group O, and Groups C and D generally differed from Group A, while Group B shared categories with both lower and higher groups (Table 3, Fig. 1). Significant linear and quadratic trends were detected ( P≤0.04 ).

Feed conversion ratio was similar during weeks 2 and 3 ( P>0.05 ) but differed during weeks 4 and 5 and overall ( P≤0.04 ; Table 4). Group D showed improved efficiency compared with Group O, while Groups B and C were intermediate, and Group A overlapped with the control. Linear and quadratic contrasts were significant ( P≤0.04 ).

The digestibility of dry matter, gross energy, ether extract, and crude protein differed among treatments ( P≤0.04 ; Table 5). Group D showed higher digestibility than Group O, while Groups B and C exhibited intermediate values, and Group A overlapped with both control and supplemented groups. Significant linear responses ( P≤0.03 ) with supporting quadratic trends were observed.

**Table 5:** Effect of different levels of blend of essential oil on nutrient digestibility (%) in broiler chicks.

	DM		GE		EE		CP	
Group 0	86.2^c^ ± 2.04		90.12^c^ ± 3.14		95.43^c^ ± 1.06		83.23^c^ ± 2.03	
Group A	88.56^b^ ± 3.14		91.43^c^ ± 2.10		96.21^bc^ ± 2.17		84.56^bc^ ± 1.08	
Group B	89.01^b^ ± 2.02		92.56^b^ ± 1.21		96.97^b^ ± 2.04		86.54^b^ ± 3.03	
Group C	91.42^ab^ ± 3.04		93.31^ab^ ± 3.06		97.12^ab^ ± 3.07		87.56^ab^ ± 2.05	
Group D	93.34^a^ ± 3.03		94.43^a^ ± 2.02		97.87^a^ ± 2.07		90.31^a^ ± 0.05	
P value	0.04		0.03		0.04		0.03	
Orthogonal contrast	Linear	0.02	Linear	0.03	Linear	0.01	Linear	0.01
	Quadratic	0.05	Quadratic	0.021	Quadratic	0.01	Quadratic	0.01

Means having different superscript in columns are significantly different ( p<0.05 ). CP, crude protein; DM, dry matter; EE, ether extract; GE, gross energy. Group O: Control group, provided with only a basal diet and no essential oil supplementation; Group A: basal diet mixed with 0.5 mL of essential oil blend per kg of feed; Group B: basal diet mixed with 1 mL of essential oil blend per kg of feed; Group C: basal diet mixed with 1.5 mL of essential oil blend per kg of feed; Group D: basal diet mixed with 2 mL of essential oil blend per kg of feed.

**Table 6:** Effect of different levels of blend of essential oil on ND (log ${}_{2})$ antibodies titer of broiler chicks.

Days	Group 0	Group A	Group B	Group C	Group D	P values	Orthogonal contrast	
7	1.2	1.4	1.4	1.5	1.6	0.06	Linear	0.33
							Quadratic	0.45
14	3.5^c^	3.6^c^	3.9^b^	4.1^ab^	4.4^a^	0.04	Linear	0.03
							Quadratic	0.02
21	4.1^c^	4.3^c^	4.6^b^	4.9^a^	5.1^a^	0.04	Linear	0.01
							Quadratic	0.01
28	4.3^c^	4.6^c^	4.8^b^	5.2^ab^	5.8^a^	0.03	Linear	0.01
							Quadratic	0.01
35	4.5^c^	4.6^c^	4.9^bc^	5.4^b^	6.1^a^	0.04	Linear	0.01
							Quadratic	0.01

Means having different superscript in rows are significantly different ( p<0.05 ). Group O: Control group, provided with only a basal diet and no essential oil supplementation; Group A: basal diet mixed with 0.5 mL of essential oil blend per kg of feed; Group B: basal diet mixed with 1 mL of essential oil blend per kg of feed; Group C: basal diet mixed with 1.5 mL of essential oil blend per kg of feed; Group D: basal diet mixed with 2 mL of essential oil blend per kg of feed.

**Table 7:** Effect of different levels of blend of essential oil on IB (log ${}_{2})$ antibodies titer of broiler chicks.

	Group 0	Group A	Group B	Group C	Group D	P value	Orthogonal contrast	
7	5454	5451	5452	5467	5465	0.06	Linear	0.66
							Quadratic	0.42
14	8879^c^	8890^c^	8997^b^	8232^ab^	8453^a^	0.04	Linear	0.03
							Quadratic	0.02
21	8845^c^	8879^c^	9102^b^	9145^a^	9343^a^	0.04	Linear	0.01
							Quadratic	0.01
28	8840^c^	8884^c^	9221^b^	9465^ab^	9543^a^	0.03	Linear	0.01
							Quadratic	0.01
35	8905^c^	8967^c^	9469^bc^	9783^b^	9956^a^	0.04	Linear	0.01
							Quadratic	0.01

Means having different superscript in rows are significantly different ( p<0.05 ). Group O: Control group, provided with only a basal diet and no essential oil supplementation; Group A: basal diet mixed with 0.5 mL of essential oil blend per kg of feed; Group B: basal diet mixed with 1 mL of essential oil blend per kg of feed; Group C: basal diet mixed with 1.5 mL of essential oil blend per kg of feed; Group D: basal diet mixed with 2 mL of essential oil blend per kg of feed.

For Newcastle disease titers, no differences were detected at day 7 ( P= 0.06). From day 14 onward, significant differences were observed ( P≤0.04 ), where Groups C and D differed from Group O, Group B was intermediate, and Group A overlapped with the control (Table 6). Linear and quadratic trends were significant ( P≤0.03 ).

**Table 8:** Effect of different levels of blend of essential oil on IBD (log ${}_{2})$ antibodies titer of broiler chicks.

Days	Group 0	Group A	Group B	Group C	Group D	P values	Orthogonal contrast	
7	3452	3463	3456	3430	3455	0.26	Linear	0.52
							Quadratic	0.41
14	1223	1242	1234	1215	1225	0.16	Linear	0.13
							Quadratic	0.12
21	2321^c^	2327^c^	2450^b^	2478^a^	2487^a^	0.04	Linear	0.01
							Quadratic	0.01
28	3245^c^	3256^c^	3376^b^	3465^ab^	3519^a^	0.03	Linear	0.01
							Quadratic	0.01
35	4154^c^	4167^c^	4454^bc^	4534^b^	4651^a^	0.04	Linear	0.01
							Quadratic	0.01

Means having different superscript in rows are significantly different ( p<0.05 ). Group O: Control group, provided with only a basal diet and no essential oil supplementation; Group A: basal diet mixed with 0.5 mL of essential oil blend per kg of feed; Group B: basal diet mixed with 1 mL of essential oil blend per kg of feed; Group C: basal diet mixed with 1.5 mL of essential oil blend per kg of feed; Group D: basal diet mixed with 2 mL of essential oil blend per kg of feed.

**Table 9:** Effect of different levels of blend of essential oil on villus histology and cecal microbial load (log_10_cfu g^−1^) of broiler chicks.

	Villus height ( µ m)		Crypt depth ( µ m)		VH : CD		*Escherichia coli*		*Lactobacillus* spp.		*Salmonella*	
Group 0	356 ± 19.7		123.61^b^ ± 8.71		4.39^a^ ± 1.46		6.65 ± 0.46		6.81 ± 0.54		6.85 ± 0.66	
Group A	337.0 ± 48.6		231.63^a^ ± 15.93		3.55^b^ ± 1.66		6.51 ± .12		6.78 ± 0.77		6.82 ± 0.28	
Group B	349.6 ± 55.5		122.34^b^ ± 4.925		2.79^ab^ ± 1.83		6.66 ± 0.22		6.59 ± 0.12		6.77 ± 0.43	
Group C	346.0 ± 51.0		125.65^b^ ± 6.77		2.86^ab^ ± 1.40		6.71 ± 0.76		6.68 ± 0.98		6.91 ± 0.21	
Group D	351.0 ± 67.0		133.01^b^ ± 7.89		2.57^ab^ ± 1.92		6.3 ± 0.98		6.71 ± 0.67		6.88 ± 0.77	
P value	0.331		0.451		0.777		0.068		0.12		0.44	
Orthogonal contrast	Linear	0.12	Linear	0.33	Linear	0.76	Linear	0.56	Linear	0.11	Linear	0.89
	Quadratic	0.25	Quadratic	0.012	Quadratic	0.44	Quadratic	0.33	Quadratic	0.71	Quadratic	0.66

Means in the same column with different superscripts are significantly different ( p<0.05 ). Group O: Control group, provided with only a basal diet and no essential oil supplementation; Group A: basal diet mixed with 0.5 mL of essential oil blend per kg of feed; Group B: basal diet mixed with 1 mL of essential oil blend per kg of feed; Group C: basal diet mixed with 1.5 mL of essential oil blend per kg of feed; Group D: basal diet mixed with 2 mL of essential oil blend per kg of feed.

A similar pattern occurred for infectious bronchitis titers: no difference at day 7 ( P= 0.06), but at days 14–35, Groups C and D differed from Group O, Group B showed intermediate separation, and Group A overlapped with lower groups ( P≤0.04 ; Table 7).

Infectious bursal disease titers did not differ at days 7 and 14 ( P>0.05 ). At days 21–35, Groups C and D showed higher responses than Group O, Group B was intermediate, and Group A overlapped statistically with the control ( P≤0.04 ; Table 8). Linear and quadratic contrasts were significant ( P= 0.01).

No significant differences were detected among Groups O, A, B, C, and D for villus height, crypt depth, villus height-to-crypt depth ratio, or cecal microbial populations ( P>0.05 ; Table 9). A quadratic trend for crypt depth was observed ( P= 0.012), suggesting a non-linear response.

## 4 Discussion

In this study, feed intake, weight gain, and FCR were significantly improved in broilers supplemented with 2 mL kg^−1^ of the EO blend. These findings support earlier reports that EO blends can enhance growth performance by improving digestive efficiency and modulating immune responses. The higher feed intake recorded in EO-supplemented groups aligns with previous observations that essential oils may stimulate appetite and increase diet palatability (Khan et al., 2023). This increased intake translated into consistent improvements in body weight, particularly in the 2 mL kg^−1^ group, which showed the most consistent performance advantages under the present experimental conditions. These results are in agreement with the findings of Hafeez et al. (2020a), who demonstrated that essential oil blends enhance nutrient absorption and support improved growth metrics.

The improvement in FCR observed in this experiment likely reflects enhancements in nutrient digestibility and immune function, both of which contribute to greater feed efficiency. Essential oils have been reported to increase digestive enzyme activity and reduce oxidative stress (Brenes and Roura, 2010), mechanisms that likely contributed to the better conversion efficiency observed in the higher-dose EO group.

The progressive enhancement of nutrient digestibility with increasing levels of EO supplementation further supports the functional benefits of essential oils in broiler nutrition. Previous findings suggest that essential oils stimulate digestive enzyme secretion, improve gut microbial balance, and reduce pathogenic bacterial load, thereby supporting more efficient nutrient breakdown and absorption (Hafeez et al., 2020a). Phenolic compounds such as thymol, carvacrol, and eugenol – derived from oregano, thyme, and cinnamon – may have contributed to these improvements through their antimicrobial and antioxidative actions. In this study, the most substantial improvements in DM, CP, EE, and GE digestibility occurred in birds supplemented with 2 mL kg^−1^ EO.

Despite these notable enhancements in growth performance, nutrient digestibility, and immune responses, the essential oil blend did not elicit significant changes in villus morphology or cecal microbiota composition. This divergence suggests that the observed performance and immunity benefits may have been mediated by functional biochemical mechanisms rather than structural changes within the intestinal mucosa (Ma et al., 2024). The unchanged cecal microbiota also suggest that the EO blend may have influenced microbial activity rather than altering the microbial community structure detectable through conventional methods. Similar outcomes have been reported where EOs improved digestive function and immunity without significant histomorphological alterations (Nahed et al., 2020; Khan et al., 2023). Thus, the lack of villus or microbiota changes in the present trial should be interpreted as an indication of the functional, rather than structural, enhancement of gut physiology.

The marked improvements in immune responses further highlight the benefits of EO supplementation. Birds receiving 2 mL kg^−1^ displayed the highest antibody titers against ND, IB, and IBD, consistent with the well-documented immunomodulatory effects of essential oils (Su et al., 2021). These effects may be attributed to bioactive compounds that stimulate macrophage activity, enhance lymphocyte proliferation, and promote cytokine production (Brenes and Roura, 2010; Hafeez et al., 2020a; Khan et al., 2023). Components such as carvacrol, thymol, cinnamaldehyde, capsicum oleoresin, and allicin have been shown to enhance GALT activation, reduce oxidative stress, and promote innate and adaptive immune responses (Spisni et al., 2020; Puvača et al., 2015; Cui et al., 2024). The sustained increase in antibody titers in the higher-dose group indicates a robust and dose-dependent immunological enhancement, which likely contributed to improved overall health and resilience.

Overall, the essential oil blend at 2 mL kg^−1^ produced the most consistent improvements in growth, feed efficiency, nutrient digestibility, and immune function under the conditions of this study. Importantly, these benefits occurred without corresponding changes in villus morphology or cecal microbiota, suggesting that essential oils primarily exerted their effects through metabolic, enzymatic, and immunological pathways rather than through structural gut modifications. Future studies using metagenomics, metabolomics, and advanced mucosal imaging may help to clarify whether EO blends influence microbial activity or epithelial signaling pathways that are not detectable through routine histology.

## 5 Conclusion

Dietary supplementation with essential oil blends, particularly at 2 mL kg^−1^ feed, improved feed intake, body weight gain, feed efficiency, nutrient digestibility, and humoral immune responses in broilers, without affecting intestinal morphology or cecal microbial populations.

## Data Availability

The data are available on request.
